# Supplementary material for: Smooth Interpolating Curves with Local Control and Monotone Alternating Curvature
Source: Comput Graph Forum. 2022 Oct 6;41(5):25–38. doi: 10.1111/cgf.14600 (PMC9827861; doi:10.1111/cgf.14600)
Supplement: Supplementary file 1 — Supplement Material [file CGF-41-25-s001.zip › Local-Smooth-Interpolating-MonoCurvature/extern/clothoids/submodules/quarticRootsFlocke/docs/api-cpp/root.html]

C++ API — Quartic Roots v1.0.0

### Navigation

- index
- toc
- next
- previous
- Quartic Roots »
- C++ API

# C++ API¶

## Class Hierarchy¶

- - Namespace PolynomialRoots
    - Class Cubic
    - Class Quadratic
    - Class Quartic

## File Hierarchy¶

- - File PolynomialRoots-1-Quadratic.cc
  - File PolynomialRoots-2-Cubic.cc
  - File PolynomialRoots-3-Quartic.cc
  - File PolynomialRoots-Jenkins-Traub.cc
  - File PolynomialRoots-Utils.cc
  - File PolynomialRoots-Utils.hh
  - File PolynomialRoots.hh

## Full API¶

### Namespaces¶

- Namespace PolynomialRoots
  - Detailed Description
  - Classes
  - Functions
  - Typedefs

### Classes and Structs¶

- Class Cubic
  - Class Documentation

- Class Quadratic
  - Class Documentation

- Class Quartic
  - Class Documentation

### Functions¶

- Function PolynomialRoots::roots
  - Function Documentation

### Typedefs¶

- Typedef PolynomialRoots::complexType
  - Typedef Documentation

- Typedef PolynomialRoots::indexType
  - Typedef Documentation

- Typedef PolynomialRoots::valueType
  - Typedef Documentation

### Quick search

### Table of Contents

- Quartic Roots Solver
- C++ API
  - Class Hierarchy
  - File Hierarchy
  - Full API

«
hide menu

menu
sidebar
»

### Navigation

- index
- toc
- next
- previous
- Quartic Roots »
- C++ API

© Copyright 2021, Enrico Bertolazzi.
Created using Sphinx 4.2.0.
